# Rebound hypercalcemia after denosumab cessation during follow-up after surgical treatment for parathyroid carcinoma: case report and literature review

**DOI:** 10.20945/2359-4292-2024-0035

**Published:** 2024-10-17

**Authors:** Lisa Schmitt, Verena Theiler-Schwetz, Patrick Sadoghi, Christian Trummer, Stefan Pilz

**Affiliations:** 1 Medical University of Graz Division of Endocrinology and Diabetology Department of Internal Medicine Graz Austria Division of Endocrinology and Diabetology, Department of Internal Medicine, Medical University of Graz, Graz, Austria; 2 Medical University of Graz Department of Orthopaedics and Trauma Graz Austria Department of Orthopaedics and Trauma, Medical University of Graz, Graz, Austria

**Keywords:** Hypercalcemia, denosumab, primary hyperparathyroidism, parathyroid carcinoma, rebound

## Abstract

Denosumab is a potent antiresorptive medication, commonly used in the treatment of osteoporosis, as well as in a variety of other diseases. Potential adverse rebound effects after its cessation include a loss in bone mineral density and an increased risk of osteoporotic fractures. Hypercalcemia is a less frequently reported rebound phenomenon after denosumab discontinuation, that may pose a diagnostic challenge to physicians as a rare non-parathyroid hormone (PTH) dependent cause of hypercalcemia. In our case, a 47-year-old male presented with rebound hypercalcemia after denosumab cessation during follow-up after surgical treatment for parathyroid carcinoma. This non-PTH-dependent hypercalcemia resolved after re-initiation of denosumab. We performed a systematic literature review on rebound hypercalcemia after denosumab cessation and identified 52 individual patient cases. Children appear to be more prone to developing rebound hypercalcemia, which could be attributed to their higher baseline bone turnover, underlying conditions, or denosumab dosage regimens. In most cases, patients initially presented with acute and often severe symptoms of hypercalcemia that occur from 1.75 to 9 months after denosumab cessation (4 to 9 months in adults). Most effective treatment approaches to sufficiently decrease serum calcium levels were bisphosphonates or re-administration of denosumab. A watch and wait strategy may be sufficient in asymptomatic cases, which are less common and probably underdiagnosed. Subsequent antiresorptive treatment after denosumab cessation, which is a common practice in osteoporosis treatment, may reduce the risk of rebound hypercalcemia. As denosumab is a frequently used drug in patients with advanced malignant diseases and rebound hypercalcemia with low PTH levels may raise the suspicion for skeletal metastases, awareness of this rebound effect may be for particular relevance in such settings.

## INTRODUCTION

Denosumab is a human monoclonal antibody against the receptor activator of NF-κB ligand (RANKL), that inhibits osteoclast activity and bone resorption by binding to soluble or membrane-bound human RANKL, preventing it from binding to its receptor, RANK (1). In consequence, it leads to a reduced risk of vertebral, nonvertebral and hip fractures in osteoporotic patients (2).

Hypercalcemia is a common phenomenon in clinical practice (3,4). In approximately 90% of all cases, it occurs due to primary hyperparathyroidism (PHPT) or a malignant condition. In the remaining 10% of cases, causes are relatively diverse and include granulomatous diseases, vitamin D intoxication, endocrine disorders, medication-related causes and renal diseases, among other rarer causes (3,4). Drug induced hypercalcemia is commonly related to the use of thiazide diuretics or lithium, in less frequent cases also to vitamin A supplements or recombinant human parathyroid hormone (rhPTH) (3,5). In contrast, denosumab-associated hypercalcemia after cessation of the drug is rarely taken into account in the differential diagnosis of elevated serum calcium levels in clinical practice.

In comparison, other rebound effects after discontinuing denosumab treatment are well established (6). Relatively soon after the cessation of treatment, typically around 9 months after the last injection on average, there is an increase in bone turnover markers. Their concentration is often significantly higher than before treatment. Furthermore, there is a decrease in bone mineral density on average after 6 months, which is also associated with an increased risk of vertebral and other osteoporotic fractures (6).

We report the rare case of rebound hypercalcemia following postoperative discontinuation of denosumab therapy in a patient who underwent parathyroidectomy due to parathyroid carcinoma. Additionally, we have performed a systematic literature review to summarize the current knowledge on this phenomenon, in order to raise awareness in clinical practitioners and provide guidance on its management.

## CASE REPORT

In October 2018, a 47-year-old male patient was referred to our outpatient clinic of the Division of Endocrinology & Diabetology at the Medical University of Graz, Austria, due to recurrent severe hypercalcemia.

The patient had previously been under nephrological care due to chronic kidney disease (current CKD stage 3b A3, initial diagnosis in 2012), attributed to the abusive use of analgetic drugs in the past.

In October 2017, he had been hospitalized due to a serum calcium level of 3.0 mmol/L (or 12 mg/dL;reference range 2.10 to 2.55 mmol/L or 8.4 to 10.2 mg/dL, respectively) that had been elevated long after cessation of calcium supplementation. Recurrent episodes of pancreatitis in the past had been described. Further laboratory evaluation had shown a serum phosphate of 0.76 mmol/L (or 2.35 mg/dL; reference range 0.87 to 1.45 mmol/L or 2.69 to 4.49 mg/dL, respectively) and a parathyroid hormone (PTH) level of 58 pg/mL (reference range 15 to 65). Malignancy as a possible cause of hypercalcemia had not been found. After treatment with intravenous hydration, furosemide and zoledronic acid, serum calcium had decreased to 2.67 mmol/L (10.68 mg/dL) at discharge.

In the following year, the patient had had further episodes of recurrent severe hypercalcemia that were treated with denosumab and cinacalcet. Denosumab was first administered at a dose of 120 mg in January 2018 and initially led to a reduction in serum calcium levels paralleled by an improvement in kidney function parameters. In May 2018, monthly doses of 120 mg denosumab were initiated due to a recurrence of severe hypercalcemia.

**Figure 1:**
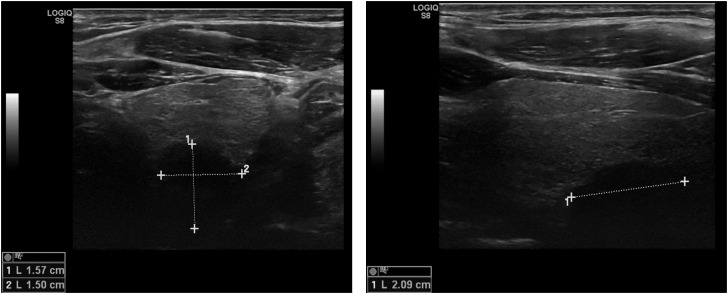
Ultrasound of the thyroid gland examination at first clinical presentation.

As no sustained and sufficient control of hypercalcemia could be achieved by the aforementioned treatment and the underlying cause remained unclear for the treating physicians, the patient was referred to our outpatient clinic in October 2018. In our initial laboratory test, a total calcium level of 3.68 mmol/L (14.72 mg/dL), an ionized calcium level of 1.97 mmol/L (7.88 mg/dL) and a PTH concentration of 375.8 pg/mL were detected, consistent with the diagnosis of PHPT. Serum creatinine was elevated at 4.22 mg/dL and thyroid hormones were within the reference range. Ultrasound examination of the thyroid gland was unremarkable, but a large hypoechoic nodule, suspicious for a parathyroid tumor, was observed on the left caudal and dorsal side of the thyroid gland (Figure 1).

In consideration of the patient's medical history and the latest findings, there was a clear indication for parathyroidectomy and the surgeon was informed that there was a suspicion of parathyroid carcinoma. Preoperatively, further administration of denosumab was critically evaluated due to the risk of postoperative hypocalcemia but had to be continued as other calcium-lowering treatment alternatives (cinacalcet 2x daily 90 mg, calcitonin 100 IU 2x daily subcutaneously) had been exhausted. Parathyroidectomy was performed in November 2018 at our surgical department. Postoperatively, there was a significant decrease in PTH and calcium levels, which remained within the upper range of normal without the need for replacement therapy. Histological examination of the surgical specimen revealed evidence of a parathyroid carcinoma infiltrating the thyroid gland and reaching the surgical margin (R1). Consequently, the patient was referred for hemithyroidectomy with neck dissection, which was successfully performed in January 2019. Subsequent histological examination of all tissues showed no evidence of malignancy.

We scheduled a follow-up appointment with the patient in our endocrine outpatient clinic two months after surgery. During this time, the patient did not receive any supplementation therapy. His chronic medications at this point only included atorvastatin and ezetimibe (due to hyperlipidemia), pantoprazole, allopurinol (due to hyperuricemia) and triazolam (due to sleep disturbances). Laboratory results again revealed an elevated total calcium level of 3.08 mmol/L (12.32 mg/dL) and an ionized calcium level of 1.59 mmol/L (6.36 mg/dL). PTH was slightly decreased at 13.5 pg/mL, as was the 25-hydroxyvitamin D level. Additionally, ß-crosslaps (1.14 ng/mL) and N-terminal propeptide of type I collagen (84.6 ng/mL) were elevated. The cause of recurrent hypercalcemia was unclear, particularly since there was no evidence of PTH-triggered hypercalcemia due to low PTH levels.

For further investigation, the patient was readmitted to our hospital in April 2019. To rule out recurrence or metastases of the pre-existing carcinoma as potential causes of hypercalcemia, a whole-body PET-CT was conducted. However, no evidence of malignancy could be found in this examination. Thus, a calcium rebound after discontinuation of denosumab therapy was primarily suspected as the triggering mechanism. Consequently, an additional 60 mg of denosumab were administered during the hospital stay. The patient was then discharged home under close electrolyte monitoring by his general practitioner.

During the following visits at our outpatient clinic, he presented in good general condition and reported good well-being. Calcium and PTH levels were within the normal range without any substitution therapy. Kidney function slightly improved to a maximum eGFR of 24.75 ml/min in June 2020 and have remained stable since then. A summary of the patient's laboratory test results is shown in Table 1. A genetic test for the presence of familial PHPT syndromes yielded unremarkable results. Treatment with denosumab was continued every three months at a dose of 120 mg. Serum calcium levels have remained within the normal range without requirement for any supplementation. Follow-up ultrasound of the thyroid and parathyroid glands have also shown no signs of disease recurrence. The patient continues to undergo regular check-ups at our outpatient clinic.

**Table 1:** Summary of the patient's laboratory test results over time

Time of measurement	Serum Ca-level	Ionized Ca-level	Serum phosphorus level	Creatinine/eGFR	PTH concentration	Denosumab treatment
Oct 2017	2.67 to 3.0 mmol/L (10.68 to 12 mg/dL)	NA	0.76 mmol/L (2.35 mg/dL)	3.04 mg/dL/ 23 mL/min	58 pg/mL (reference range 15 to 65)	No (but single administration of zoledronic acid)
Nov 2017	2.45 mmol/L (9.8 mg/dL)	NA	NA	NA/23 mL/min	NA	No
Jan 2018	3.09 mmol/L (12.36 mg/dL)	1.63 mmol/L (6.52 mg/dL)	NA	NA	NA	120 mg administered, administration every 6 months planned
Feb 2018	2.69 mmol/L (10.76 mg/dL)	1.42 mmol/L (5.68 mg/dL)	NA	NA/34 mL/min	NA	No
May 2018	3.92 mmol/L (15.68 mg/dL)	2.01 mmol/L (8.04 mg/dL)	NA	3.51 mg/dL/NA	NA	120 mg administered, monthly administration planned
Aug 2018	NA	2.09 mmol/L (8.36 mg/dL)	NA	NA	NA	120 mg monthly
Sep 2018	3.3 mmol/L (13.2 mg/dL)	1.73 mmol/L (6.92 mg/dL)	0.97 mmol/L (3.00 mg/dL)	3.48 mg/dL/ 20 mL/min	219 pg/mL	120 mg monthly
Oct 2018	3.68 mmol/L (14.72 mg/dL)	1.97 mmol/L (7.88 mg/dL)	1.04 mmol/L (3.22 mg/dL)	4.22 mg/dL/ 15.6 mL/min	375.8 pg/mL	120 mg monthly
**Parathyroidectomy performed in November 2018, last administration of denosumab 4 weeks before surgery.**						
Dec 2018	2.64 mmol/L (10.56 mg/dL)	1.38 mmol/L (5.52 mg/dL)	1.51 mmol/L (4.69 mg/dL)	4.82 mg/dL/13.3 mL/min	32.4 pg/mL	No
**Hemithyroidectomy with Neck dissection in January 2019.**						
Apr 2019	3.08 mmol/L (12.32 mg/dL)	1.59 mmol/L (6.36 mg/dL)	1.28 mmol/L (3.97 mg/dL)	3.65 mg/dL/ 18.5 ml/min	13.5 pg/mL	Administration of 60 mg
Jul 2019	2.46 mmol/L (9.84 mg/dL)	1.26 mmol/L (5.04 mg/dL)	1.16 mmol/L (3.60 mg/dL)	4.34 mg/dL/ 15.0 mL/min	32.5 pg/mL	No
Sep 2019	3.0 mmol/L (12 mg/dL)	NA	NA	NA/14 mL/min	NA	120 mg every 3 months
Dec 2019	2.39 mmol/L (9.56 mg/dL)	1.22 mmol/L (4.88 mg/dL)	0.69 mmol/L (2.15 mg/dL)	3.48 mg/dL/ 19.5 mL/min	35.0 pg/mL	120 mg every 3 months
Jun 2020	2.57 mmol/L (10.28 mg/dL)	1.3 mmol/L (5.2 mg/dL)	0.92 mmol/L (2.86 mg/dL)	2.85 mg/dL/ 24.8 mL/min	12.5 pg/mL	120 mg every 3 months
Sep 2021	2.58 mmol/L (10.32 mg/dL)	1.27 mmol/L (5.08 mg/dL)	1.02 mmol/L (3.16 mg/dL)	3.03 mg/dL/ 22.8 mL/min	21.9 pg/mL	120 mg every 3 months

NA: data not available.

### Literature review

A PubMed search was performed on May 09, 2023 using the search terms "denosumab rebound", "denosumab cessation", "denosumab discontinuation" and "denosumab interruption" together with "hypercalcemia", respectively. By screening the abstracts of all search results, 32 publications describing cases of rebound hypercalcemia after denosumab cessation could be found, including 52 individual patient cases (7–38).

The following data were extracted from each of the retrieved cases: patient sex, patient age at the time of first denosumab administration, indication for denosumab therapy, total duration of therapy, dosage and interval of therapy, time interval between the last administration of denosumab and the occurrence of hypercalcemia, total calcium level, symptoms of hypercalcemia and treatment of hypercalcemia. We assumed no treatment if it was not described. If the relevant data were not precisely specified, they were estimated, if possible, based on the available information. Duplicate patient cases were removed and if a publication provided no clinical information on a case, it was excluded from the an individual cases is shown in Tables 2 and 3.

**Table 2:** Characteristics of children with rebound hypercalcemia after cessation of denosumab treatment

Case Nr.	Reference Nr.	Sex	Age (years)	Indication for denosumab treatment	Treatment duration (months)	Treatment frequency	Cumulative dose/dose per year (mg)	Time to hypercalcemia (months)	Hypercalcemic symptoms	Serum Ca- level (mmol/L, mg/dL in [])
11	(32)	m	1	Osteogenesis Imperfecta Type VI	30	Every 2-6 months	NA	1.75	NA	Ionized Ca: 1.62 [6.48]
47	(37)	m	2	Central Giant Cell Granuloma	14	Monthly with 4 weeks loading	NA	4	Yes	3.4 [13.6]
10	(32)	f	2	Osteogenesis Imperfecta Type VI	24	Every 3 months	NA	3	NA	Ionized Ca: 1.54 [6.16]
20	(14)	m	3	Noonan Syndrome	8	Monthly	225/335	2	Yes	3.39 [13.56]
7	(18)	f	5	Juvenile Xanthogranuloma	3-(7)-3	Monthly with 4 weeks loading	1,200/1,111	7	Yes	3.28 [13.12]
29	(31)	m	6	Aneurysmal Bone Cyst	12	Monthly with 4 weeks loading	900/900	3	Yes	4.14 [16.56]
33	(10)	f	6	Central Giant Cell Granuloma	<24	Monthly with 4 weeks loading	<1,560/n.a.	NA	Yes	NA
15	(13)	m	7	Aneurysmal Bone Cyst	12	Monthly with 4 weeks loading	840/840	6	NA	NA
16	(27)	m	8	Aneurysmal Bone Cyst	12	Monthly with 4 weeks loading	NA	5	NA	NA
36	(11)	m	8	Aneurysmal Bone Cyst	12-(9)-30	Monthly with 4 weeks loading, tapering in the end	NA	1.5-6 (3 hypercalcemic episodes described)	Yes	3.7 [14.8]
2	(16)	f	8	Juvenile Paget Disease	1.5	Only 2 doses administered	NA	1.75	Yes	4.07 [16.28]
22	(14)	m	8	Noonan Syndrome	7.5	Every 1-2.5 months	210/336	2.5	Yes	3.64 [14.56]
48	(37)	m	9	Central Giant Cell Granuloma	13	Monthly with 4 weeks loading	NA	4.5	Yes	3.66 [14.64]
3	(25)	f	9	Central Giant Cell Granuloma	18	Monthly with 4 weeks loading	2,520/1,680	NA	NA	2.67 [10.68]
37	(22)	f	9	Central Giant Cell Granuloma	10	Monthly with 4 weeks loading	1,560/1,879	6.5	Yes	2.35 [9.4]
25	(19)	m	9	Cherubism	6	Every 3-4 weeks	960/1,920	6	NA	NA
1	(8)	m	9	Fibrous Dysplasia/McCune-Albright-Syndrome	7	Monthly	NA	2	Yes	4.5 [18.0]
51	(36)	f	10	Aneurysmal Bone Cyst	18	4 weeks loading, then every 1-2 months	NA	NA	Yes	3.37 [13.48]
40	(12)	m	10	Aneurysmal Bone Cyst	10	Monthly with 4 weeks loading	1,560/1,880	4	Yes	3.78 [15.12]
46	(37)	f	10	Central Giant Cell Granuloma	13	Monthly with 4 weeks loading	NA	5	Yes	2.88 [11.52]
6	(30)	m	10	Giant Cell Tumor of the Bone	5-(5)-4	Monthly with 4 weeks loading	1,440/1,231	4	Yes	3.79 [15.16]
4	(15)	f	10	Giant Cell Tumor of the Bone	24	Monthly with 4 weeks loading	3,240/1,620	5	Yes	4.12 16.48]
27	(31)	m	11	Aneurysmal Bone Cyst	16	4 weeks loading, then every 1-2 months	960/722	2.5	Yes	3.95 [15.8]
34	(10)	m	11	Central Giant Cell Granuloma	<24	Monthly with 4 weeks loading	<1,560/n.a.	5	Yes	NA
28	(31)	f	11	Giant Cell Tumor of the Bone	12	4 weeks loading, then every 1-2 months	840/840	3	Yes	3.93 [15.72]
50	(36)	m	12	Aneurysmal Bone Cyst	18	Monthly with 4 weeks loading	NA	NA	Yes	3.12 [12.48]
18	(35)	f	12	Aneurysmal Bone Cyst	12	Monthly with 4 weeks loading	1,680/1,680	5	Yes	3.87 [15.48]
45	(37)	f	12	Central Giant Cell Granuloma	18-(12)-20	4 weeks loading, then every 1-2 months	NA	7	No	2.74 [10.96]
35	(10)	f	12	Central Giant Cell Granuloma	<24	Monthly with 4 weeks loading	<1,560/n.a.	NA	Yes	NA
52	(36)	m	13	Aneurysmal Bone Cyst	12	Monthly with 4 weeks loading	NA	NA	Yes	3.89 [15.56]
24	(17)	m	13	Aneurysmal Bone Cyst	27	4 weeks loading, then every 1-3 months	1,554/691	6	Yes	4.04 [16.16]
39	(26)	f	13	Fibrous Dysplasia	42	Every 1-3 months	1,864/533	5	Yes	3.14 [12.56]
30	(31)	m	13	Giant Cell Tumor of the Bone	13-(30)-7	Monthly with 4 weeks loading	1,380/n.a.	3	Yes	>4 [>16]
23	(14)	m	13	Noonan Syndrome	6	Monthly	420/840	3	Yes	2.67 [10.68]
32	(7)	m	13	Primary Pediatric Osteoporosis	30	Every 3 months	600/240	3	No	3.09 [12.36]
41	(12)	m	14	Central Giant Cell Granuloma	12	Monthly with 4 weeks loading	1,500/1,500	5	Yes	3.84 [15.36]
12	(34)	f	14	Giant Cell Tumor of the Bone	15	Monthly with 4 weeks loading	2,160/1,728	6	Yes	3.4 [13.6]
13	(34)	m	15	Giant Cell Tumor of the Bone	43	Monthly with 4 weeks loading	5,520/1,542	7	Yes	3.1 [12.4]
8	(21)	NA	12-17	Aneurysmal Bone Cyst	14	Monthly with 4 weeks loading	NA	5	NA	NA
9	(21)	NA	12-17	Aneurysmal Bone Cyst	14	Monthly with 4 weeks loading	NA	5	NA	NA
21	(14)	m	17	Noonan Syndrome	>12	4 weeks loading, then every 1-3 months	>900/n.a.	>3	Yes	2.87 [11.48]
44	(28)	NA	<18	Central Giant Cell Granuloma	NA	Monthly	NA	NA	NA	2.71 [10.84]

NA: data not available; n.a.: not applicable. Treatment duration: Numbers in brackets illustrate treatment interruptions. In (32), only ionized Ca-levels and no total Serum-Ca levels are available.

**Table 3:** Characteristics of adults with rebound hypercalcemia after cessation of denosumab treatment

Case Nr.	Reference Nr.	Sex	Age (years)	Indication for denosumab treatment	Treatment duration (months)	Treatment frequency	Cumulative dose/dose per year (mg)	Time to hypercalcemia (months)	Hypercalcemic symptoms	Serum Ca- level (mmol/L, mg/dL in [])
42	(28)	NA	>18	Central Giant Cell Granuloma	NA	Monthly	NA	NA	No	2.61 [10.44]
43	(28)	NA	>18	Central Giant Cell Granuloma	NA	Monthly	NA	NA	No	2.61 [10.44]
14	(34)	m	40	Giant Cell Tumor of the Bone	48	Monthly with 4 weeks loading	6,120/1,530	5.5	Yes	4.27 [17.08]
31	(33)	f	42	Breast Cancer	60	Every 1-3 months	3,600/720	6	Yes	3.59 [14.36]
49	(38)	f	42	Breast Cancer	78	Every 2 months	5,040/775	8	Yes	3.29 [13.16]
38	(24)	f	43	Fibrous Dysplasia/McCune-Albright-Syndrome	24	Every 3 months	540/270	5	No	2.73 [10.92]
17	(29)	f	49	Breast Cancer	60	Every 1-3 months	3,000/600	4	Yes	3.1 [12.4]
26	(23)	f	64	Osteoporosis	69	Every 6 months	720/125	9	No	2.83 [11.32]
5	(20)	f	67	Osteoporosis	114	Every 6 months	1,200/126	6	No	3.1 [12.4]
19	(9)	f	86	Primary Hyperparathyroidism	41	Every 6 months	420/123	6-9	Yes	2.8-3.1 [11.2-12.4]

NA: data not available; n.a.: not applicable. Treatment duration: Numbers in brackets illustrate treatment interruptions.

Of the 52 patients, 42 were younger than 18 years and only 10 were adults and accordingly skeletally mature. Patient age in the adult group ranged from 40 to 86 years while in child cases, the youngest patient was 1.9 years old and the oldest was 17 years.

Sex distribution differed between the two age groups. Among children and adolescents, there were more reported cases of boys with rebound hypercalcemia (24 boys, 15 girls, unspecified sex in 3 cases). The case reports of adult patients almost exclusively described female patients (7 women, 1 man, unspecified sex in 2 cases).

Diagnoses indicating denosumab therapy differed significantly between the age groups. Children received denosumab because of aneurysmal bone cysts (n = 13), central giant cell granuloma (including Noonan syndrome, n = 15) and giant cell tumor of the bone (n = 6). Rare treatment indications were fibrous dysplasia (including McCune-Albright syndrome, n = 2), juvenile Paget's disease (n = 1), juvenile xanthogranuloma (n = 1), osteogenesis imperfecta type VI (n = 2), primary pediatric osteoporosis (n = 1) and cherubism (n = 1).

Case reports of adult patients with rebound hypercalcemia included cases of breast cancer (n = 3), osteoporosis (n = 2), and one case of PHPT. Additionally, there were two cases of central giant cell granuloma, one case of giant cell tumor of the bone, and one case of fibrous dysplasia (McCune-Albright syndrome) reported in adulthood.

Total duration of denosumab therapy before its cessation varied significantly among individual cases, ranging from 1.5 to 114 months. Overall, it was notably longer for adults compared to children, although the dosing interval was also generally longer in the adult group. The cumulatively administered dose ranged from 210 to 5,520 mg in children and from 420 to 6,120 mg in adults. In most cases, denosumab was administered in a weight-adapted manner for children, while standard dosages were used for adults. On an annual basis, children received markedly higher doses on average than adults (average dose per year 1,506 mg *vs.* 534 mg). Among the adult patients, three received the typical dose of 60 mg every 6 months, that is commonly used in osteoporosis therapy, while five were treated with higher doses.

The time interval between the last dose of denosumab and the occurrence of hypercalcemia ranged from 1.75 to 7 months in children and from 4 to 9 months in adults. In adult patients, the time gap was generally longer compared to children (mean time interval of 4.23 months in children *vs.* 6.19 months in adults).

In the majority of cases, hypercalcemia after cessation of denosumab was detected due to typical symptoms attributable to hypercalcemia, such as polyuria, polydipsia, nausea, vomiting, fatigue, muscle weakness or dizziness. Treatment approaches for this rebound hypercalcemia after denosumab cessation mostly involved intravenous hydration (n = 31), in some cases combined with loop diuretics (n = 13). However, in most cases, this therapeutic approach did not achieve sufficient control of hypercalcemia. Ultimately, the use of bisphosphonates frequently led to a satisfactory reduction and normalization in serum calcium levels. Treatment with bisphosphonates was reported in 35 cases. In some cases, denosumab was readministered, which was also usually successful in treatment of hypercalcemia (n = 12). Some treatment approaches also included the use of calcitonin (n = 8), corticosteroids (n = 6) or cinacalcet (n = 1). Only few cases of (asymptomatic) rebound hypercalcemia were self-limiting (n = 4).

In the cases of adult patients, it is not reported whether bisphosphonates were initiated after the completion of denosumab treatment to prevent rebound events, therefore we assume that the patients did not receive any antiresorptive therapy after cessation of denosumab.

Due to the highly heterogeneous patient cohort and the lack of comparability among cases, a deliberate decision was made to refrain from conducting an exploratory statistical analysis of the data.

## DISCUSSION

We present the case of a patient with long-standing and undiagnosed PHPT due to parathyroid carcinoma, in whom denosumab was administered to treat hypercalcemia before parathyroid surgery. Post-surgical follow-up examination revealed PTH-independent hypercalcemia consistent with a rebound hypercalcemia after cessation of long-term denosumab treatment. Hypercalcemia resolved after re-initiation of denosumab therapy. In addition, we performed a systematic literature review on this issue and retrieved 52 published cases with rebound hypercalcemia after denosumab cessation.

Since carcinomas of the parathyroid glands account for only about 1% of all cases of PHPT, preoperative differentiation between benign and malignant lesions poses significant clinical challenges (39,40). The main features that indicate an increased malignancy risk in hormonally active parathyroid masses are tumor size and metabolic activity of the tumor (41,42). Accordingly, lesions are considered suspicious when their size exceeds 3 cm and when patients present with high serum calcium levels (>3.0 mmol/L) (41,42). Often, markedly elevated PTH levels are observed in parathyroid carcinoma patients (42). Clinicians should refrain from parathyroid biopsy or fine needle aspirations of parathyroid lesions with high risk of malignancy due to the risk of tumor seeding (40). In cases of high suspicion for malignancy, oncological en bloc resection should be preferred over local excision in order to improve the patient's prognosis (40,42). In our case, the second surgery, a hemithyroidectomy with neck dissection, could achieve a favorable prognostic outcome with high probability.

Our patient's significantly impaired kidney function may be attributed to long-standing PHPT rather than, as initially assumed, being a result of analgetic drug abuse. However, since the exact onset and chronological sequence of the occurrence of renal insufficiency and hyperparathyroidism in our case are unknown, it is not possible to draw definite conclusions. Anyway, renal manifestations, including impaired kidney function and kidney/urinary stones, occur in 37.2% of patients who initially present with parathyroid cancer and therefore are a common diagnostic feature (39).

Regarding the patient's history of recurrent episodes of pancreatitis, a hypercalcemic etiology appears highly probable in retrospect. However, other possible causes (e.g., an ethyltoxic etiology) cannot be ruled out.

With the appearance of postoperative recurrence of high calcium levels in our patient, hypercalcemia of malignancy was initially a plausible assumption. PTH-dependent hypercalcemia indicating persistent PHPT (43) was excluded, since PTH concentrations were sufficiently suppressed. Additionally, there was no sign of malignancy in functional imaging studies. Therefore, a rebound phenomenon after discontinuation of denosumab was ultimately the most likely explanation, justifying the re-initiation of that treatment in our patient. This was particularly unavoidable as bisphosphonate therapy was contraindicated due to advanced chronic kidney disease.

Our literature review revealed that hypercalcemia is a rarely diagnosed complication after cessation of denosumab treatment. The fact that, according to our literature research, it occurs much more frequently in children than in adults may be attributed to their higher baseline bone turnover (10,11,15,37,38,44). In line with this, high bone turnover disorders, requiring the use of denosumab in children, were frequent underlying diseases in children with rebound hypercalcemia (11,44). This could also explain why rebound hypercalcemia in children may occur earlier and even during ongoing denosumab treatment, when dosing intervals are extended.

In our analysis of the reported cases, children had, on average, also received significantly higher doses compared to adults. Generally, based on the limited number of cases, it is difficult to assess whether higher dosages of denosumab are also associated with an increased risk of rebound hypercalcemia. In adults, the effect occurred in both the common standard dose of 120 mg per year and in higher dosages, although slightly more cases were reported in whom this standard dose was exceeded.

By far the most common indications for denosumab therapy in children were central giant cell granulomas and aneurysmal bone cysts. In adults, the most frequent indication was breast cancer, albeit with only three reported cases. In any case, no condition is emerging in which rebound hypercalcemia would occur more frequently than in others.

A successful therapeutic control of rebound hypercalcemia was ultimately achieved in most cases only after administration of bisphosphonates or re-initiation of denosumab. Exclusive treatment with hydration or loop diuretics was generally not effective. In cases of symptomatic rebound hypercalcemia following discontinuation of denosumab, early administration of bisphosphonates or re-admission of denosumab may therefore be considered by clinicians to achieve rapid serum calcium normalization. In asymptomatic rebound hypercalcemia, the course was often reported as self-limiting, thus an initial watch and wait strategy might be appropriate in these cases.

Considering all reported cases, we must also note that it is not entirely clear whether there is indeed a pure rebound hypercalcemia evident or if there are other underlying mechanisms of hypercalcemia present. E.g., in case 26 (23), there is documented parathyroid hyperplasia with PHPT, and at the time of elevated calcium levels, PTH was within the normal range and not elevated as expected, thus indicating the presence of PHPT rather than rebound hypercalcemia. In the case report by Camponovo and cols. (9), PHPT is also described as an additional factor contributing to hypercalcemia.

The fact that most described cases of rebound hypercalcemia are associated with typical hypercalcemic symptoms could be an indication that this phenomenon is underdiagnosed, and mild, asymptomatic rebound hypercalcemia (which might be much more common than severe, symptomatic cases) is often not recognized as such.

In differential diagnosis of hypercalcemia, the described rebound effect is, of course, a relatively rare underlying condition. Nevertheless, it should be considered as a possible explanation in patients with a suitable medical history and after ruling out other causes, in order to subsequently initiate appropriate treatment. As denosumab is a frequently used drug in patients with advanced malignant diseases, and rebound hypercalcemia with low PTH levels may raise the suspicion for skeletal metastases, awareness of this rebound effect may be of particular relevance for further patient management in such settings.

In general, denosumab is very effective in treating hypercalcemia and we refer the reader to excellent publications on this issue (45,46). An important clinical consideration is that it usually takes a few days after denosumab application before a significant decline in calcium concentrations is observed. In the treatment of hypercalcemia of malignancy in adults, a recent Endocrine Society Guideline recommends the use of denosumab over intravenous bisphosphonates (46). However, data are insufficient at the moment to draw firm conclusions on this issue in children. As rebound hypercalcemia can be a serious adverse effect of denosumab withdrawal particularly in children, an individualized risk-benefit assessment should be conducted prior to the initiation of treatment. With regard to osteoporosis and some non-osteoporotic conditions in children, several studies support the safety and efficacy of bisphosphonates as an off-label treatment. Evidence is limited on the respective use of denosumab in children, which should only be considered with precaution as an off-label treatment in advanced kidney disease, poor treatment efficacy or severe side effects of bisphosphonates, and in children with giant cell tumor of the bone (GCTB) or aneurysmal bone cysts (47). Since rebound effects characterized by increased bone turnover parameters and decreased bone mineral density with consequently increased fracture risk are known after discontinuation of denosumab, a follow-up treatment with bisphosphonates is recommended, if possible (48,49). It can be assumed that such treatment could also counteract rebound hypercalcemia, especially since in the described adult cases, apparently no patient had received bisphosphonate treatment after denosumab cessation.

In summary, our research has revealed that the occurrence of PTH-independent hypercalcemia, alongside other more commonly described rebound effects, can be a potential side effect following the discontinuation of denosumab. This appears to be significantly more frequent and potentially more severe in children compared to adults. The most effective treatment consists of administering bisphosphonates or reinitiating denosumab. In mild, asymptomatic cases, a watch-and-wait strategy may be sufficient. Awareness of this rebound effect in adult patients may be of particular relevance in settings of malignant diseases as it is an important differential diagnosis for hypercalcemia of malignancy.
